# Supplementary material for: T cell memory response to MPXV infection exhibits greater effector function and migratory potential compared to MVA-BN vaccination
Source: Nat Commun. 2025 May 10;16:4362. doi: 10.1038/s41467-025-59370-5 (PMC12065855; doi:10.1038/s41467-025-59370-5)
Supplement: Supplementary file 1 — Supplementary Information [file 41467_2025_59370_MOESM1_ESM.pdf]

Supplementary Information

Contents:

- Supplementary Figure Legends
- Supplemental Figure 1
- Supplemental Figure 2
- Supplemental Figure 3
- Supplemental Figure 4
- Supplemental Figure 5
- Supplemental Figure 6

Supplementary Figure 1. HLA coverage of mpox convalescent donors in the study The frequency of each HLA-A, HLA-B, HLA-DRB1 and HLA-DQB1 of the Mpox-convalescent individuals in this study.

Supplementary Figure 2. Ex vivo ELISpot of FEC peptide mix (A): Summary of VACV-induced memory T cell response in mpox convalescent (N=13) and healthy control (HC, N=10) participants against the influenza, Epstein-Barr virus (EBV) and human cytomegalovirus (HCMV) (FEC) peptide mix as a positive control (P=0.723) (B): Proportion of the total ELISpot T cell response for HLA-A*02:01^{+} individuals that show greater response to mega pools compared to VACV, split into response to CD4 mega pool, non-HLA-A*02 CD8 mega pool and HLA-A*02 CD8 mega pool. Data are presented as median±IQR for (A). The Mann-Whitney U-test was used for the analysis and two-tailed P values were calculated. ns=not significant, SFU=spot-forming units

Supplementary Figure 3. Representative gating strategies of flow cytometry analysis Cells were first gated on single Lymphocytes by a forward side scatter gate. After excluding dead cells, cells then were gated on (A): CD3^{+}CD4^{+} T cells or CD3^{+}CD8^{+} T cells for further analysis with activation induced markers (AIMs assay); (B): CD8^{+} T cells for further IFNγ +/-, TNFα+/-, MIP1ß+/- and CD107a+/- populations via ICS. (C): CD3^{+}CD8^{+} Tetramer^{+} T cells *for ex* vivo phenotyping gating. (D): Original sorting gate of live CD3^{+}CD8^{+}Tetramer^{+} T cells for *ex vivo* single-cell RNASeq.

Supplementary Figure 4: Representative FACS plots for killing assay. Cells were first gated on single Lymphocytes by a forward side scatter gate. Live target cells were identified as CFSE^{+}CD19^{+}7-AAD^{-} cells

Supplementary Figure 5: Quality control of scRNAseq data (A) UMAP of the scRNAseq data coloured by participant, showing a roughly equal distribution of cells in the UMAP space, independent of donor origin (B) Normalized expression of *CD3E*, *PTPRC* (CD45) and *CD8A* in the single-cell RNAseq data. N=401 and n=160 cells from convalescent and vaccinated individuals respectively.

Supplementary Figure 6. Representative gating of cytotoxic and migratory molecules expressed on ILD-Tetramer^{+}CD8^{+} T cells. (A): Cells were first gated on single Lymphocytes by a forward side scatter gate. After excluding dead cells, cells then were gated on CD3^{+}CD8^{+} T cells and CD3^{+}CD8^{+}Tetramer^{+} T cells. (B) and (C): CD44 high/low, CD49d+/-, CD29+/-, Granulysin+/- and Granzyme A+/- gating on overall CD8^{+} T cells and CD8^{+}Tetramer^{+} T cells, respectively. (D): Example of fluorescence intensity of CD44, CD49d and CD29 expressed on CD8^{+}Tetramer^{+} T cells from Convalescent and Vaccinated

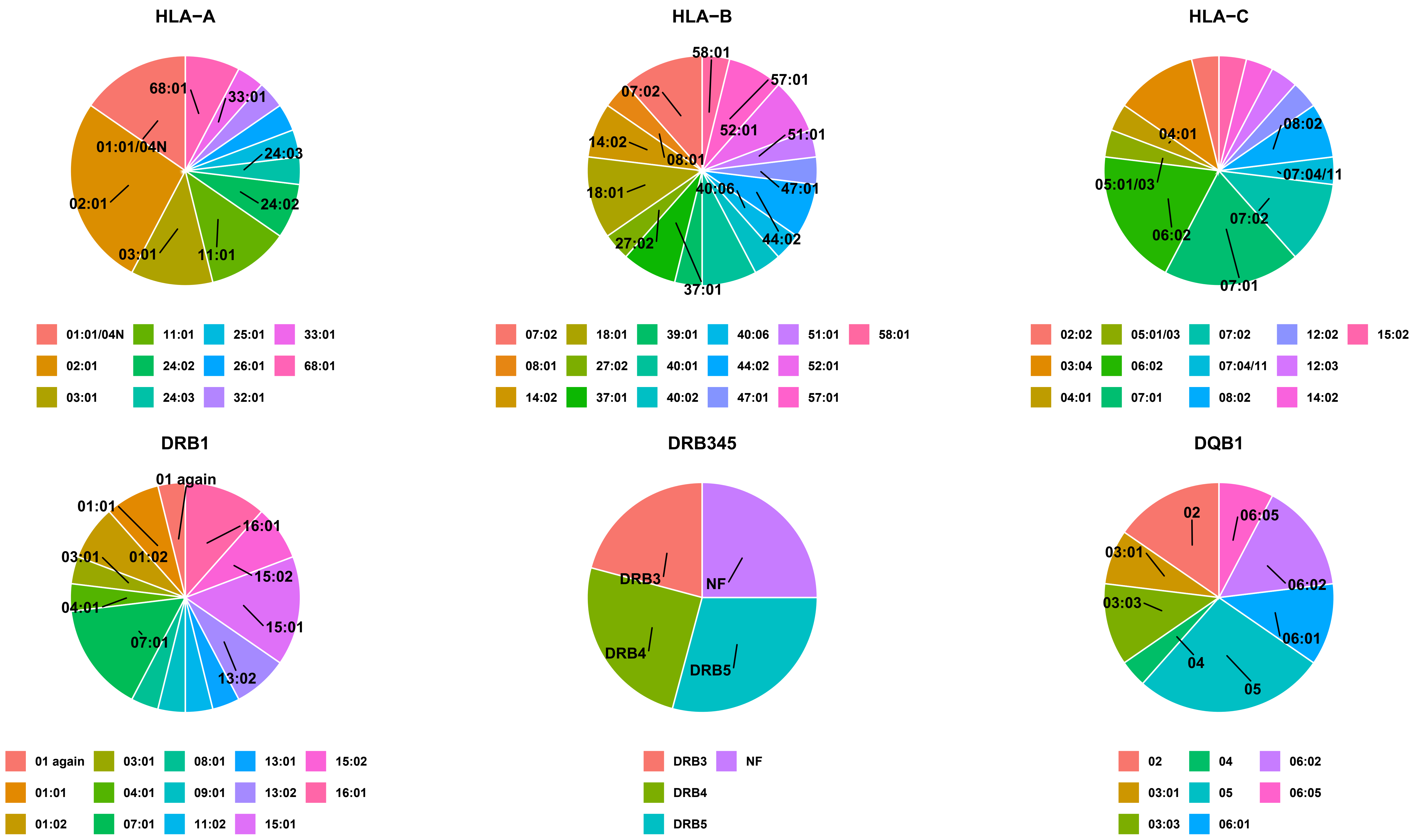

Supplementary Figure 1

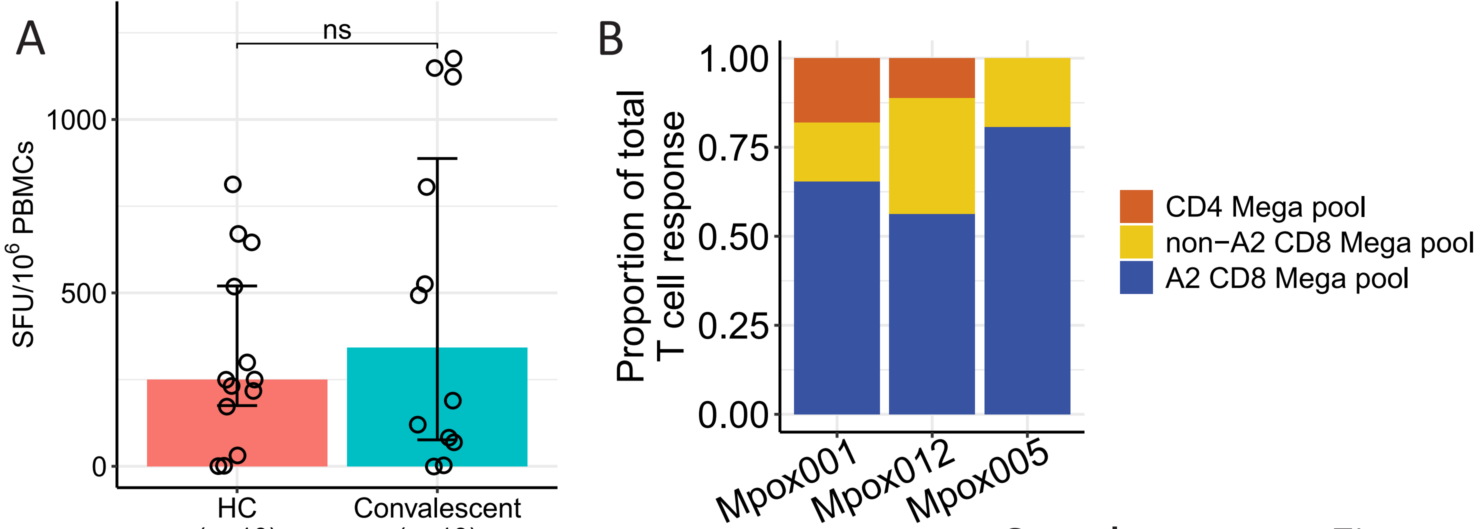

Supplementary Figure 2

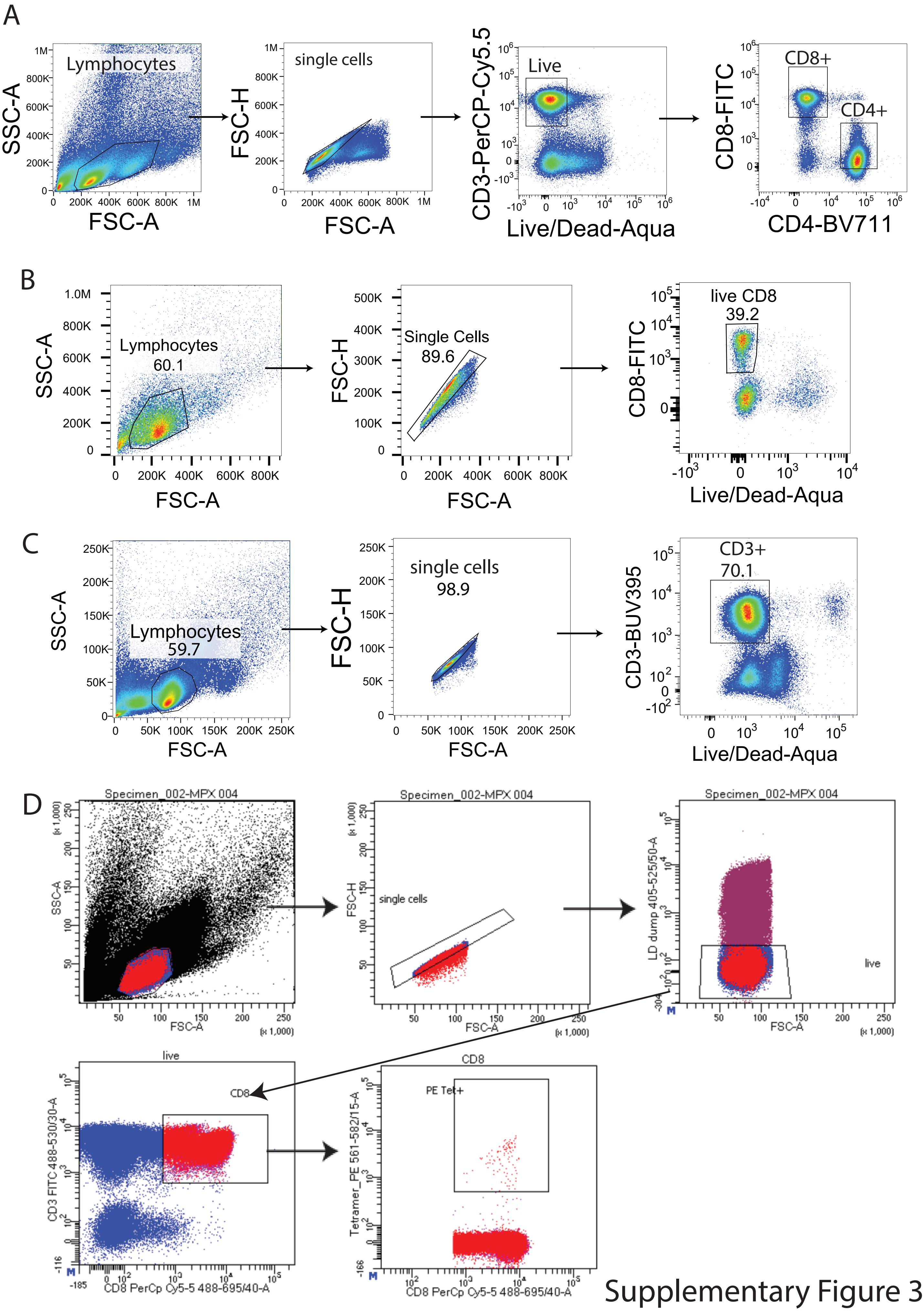

Supplementary Figure 3

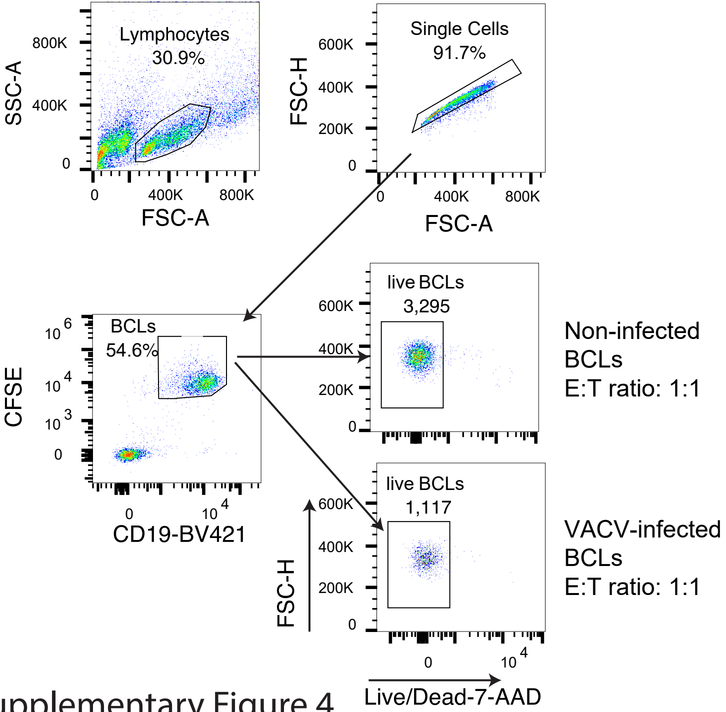

Supplementary Figure 4

Supplementary Figure 5

A

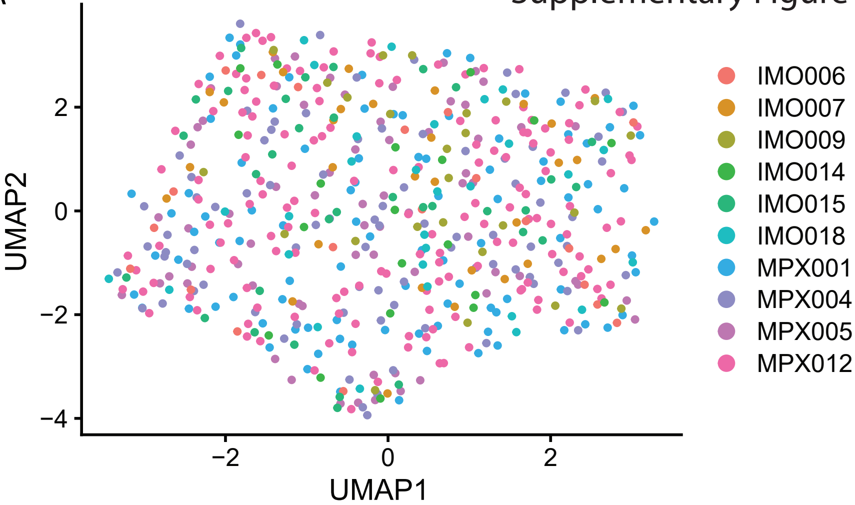

B

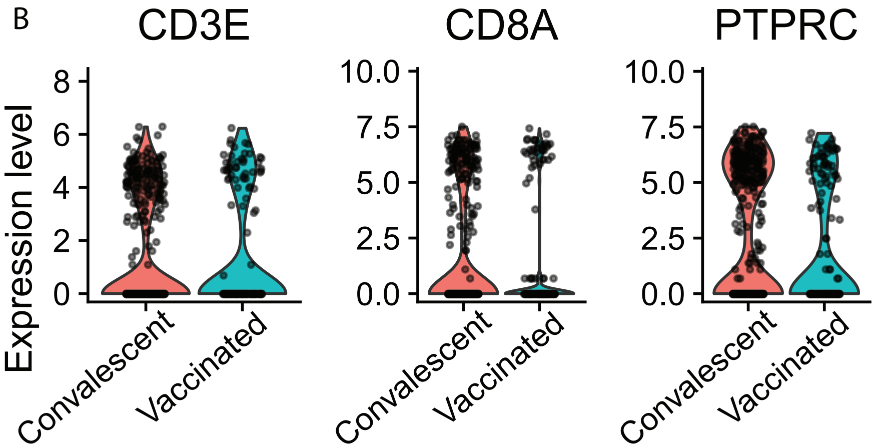

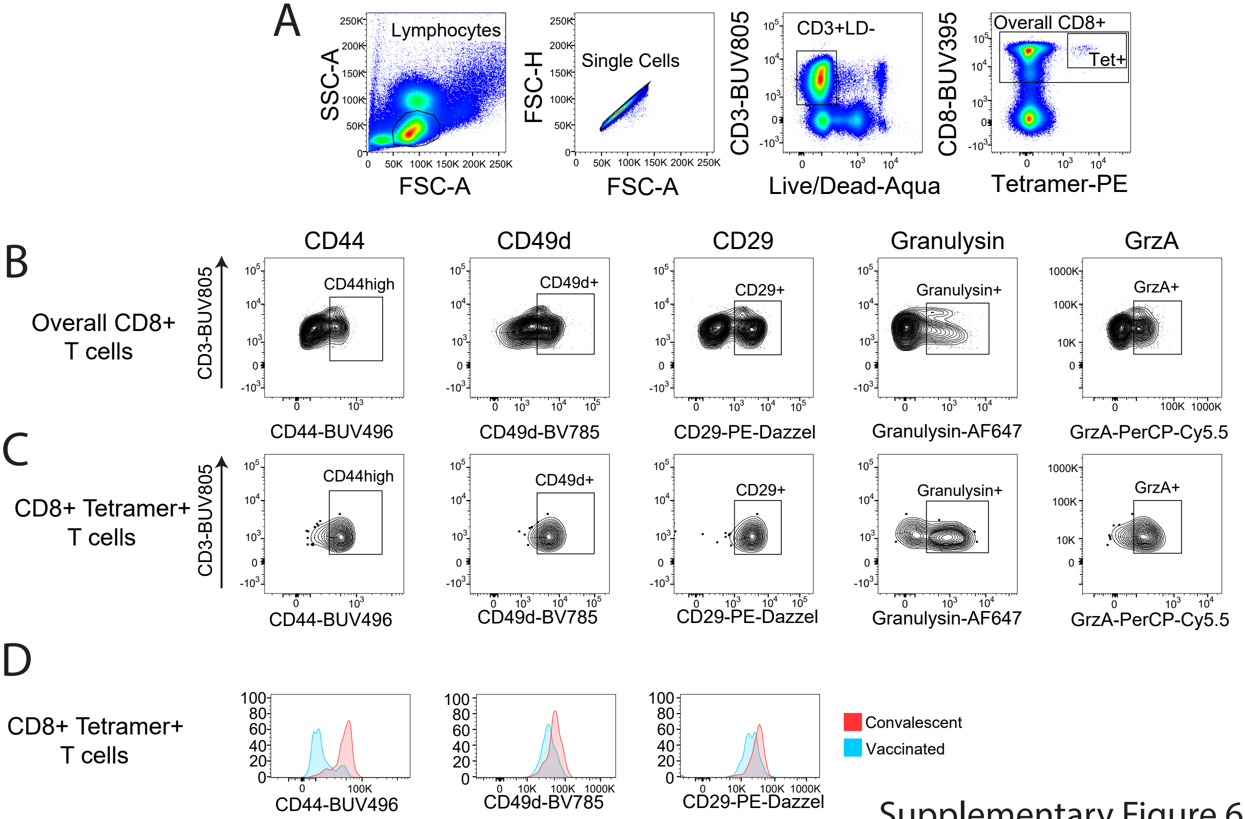

Supplementary Figure 6
