# Supplementary material for: T cell memory response to MPXV infection exhibits greater effector function and migratory potential compared to MVA-BN vaccination
Source: Nat Commun. 2025 May 10;16:4362. doi: 10.1038/s41467-025-59370-5 (PMC12065855; doi:10.1038/s41467-025-59370-5)
Supplement: Supplementary file 2 — Description of Additional Supplementary Files [file 41467_2025_59370_MOESM2_ESM.pdf]

Description of Additional Supplementary Files

Supplementary Data 1. HLA typing of individuals in the Mpox-convalescent cohort

Supplementary Data 2. Vaccinated cohort characteristics

Clinical characteristics of the vaccinated cohort. NA=not available

Supplementary Data 3. Individual peptides in the CD8 and CD4 mega-pools

Individual peptides synthesized for use in the CD8 and CD4 mega-pools, with the synthesized sequence (Sequence in MPXV) and homologous sequence in vaccinia virus (Sequence in VACV). *bold and underlined residues indicate substitutions from the VACV genome to the MPXV genome

Supplementary Data 4. Genes used to generate module scores for the scRNAseq analysis

Supplementary Data 5. Antibody panel for AIMs assay
