# Supplementary material for: T cell memory response to MPXV infection exhibits greater effector function and migratory potential compared to MVA-BN vaccination
Source: Nat Commun. 2025 May 10;16:4362. doi: 10.1038/s41467-025-59370-5 (PMC12065855; doi:10.1038/s41467-025-59370-5)
Supplement: Supplementary file 4 — Reporting Summary [file 41467_2025_59370_MOESM4_ESM.pdf]

nature portfolio

Corresponding author(s): Tao Dong

Last updated by author(s): Mar 12, 2025

Reporting Summary

Nature Portfolio wishes to improve the reproducibility of the work that we publish. This form provides structure for consistency and transparency in reporting. For further information on Nature Portfolio policies, see our Editorial Policies and the Editorial Policy Checklist.

Statistics

For all statistical analyses, confirm that the following items are present in the figure legend, table legend, main text, or Methods section.

n/a	Confirmed	
☐	☒	The exact sample size (*n*) for each experimental group/condition, given as a discrete number and unit of measurement
☐	☒	A statement on whether measurements were taken from distinct samples or whether the same sample was measured repeatedly
☐	☒	The statistical test(s) used AND whether they are one- or two-sided<br>*Only common tests should be described solely by name; describe more complex techniques in the Methods section.*
☐	☒	A description of all covariates tested
☐	☒	A description of any assumptions or corrections, such as tests of normality and adjustment for multiple comparisons
☐	☒	A full description of the statistical parameters including central tendency (e.g. means) or other basic estimates (e.g. regression coefficient) AND variation (e.g. standard deviation) or associated estimates of uncertainty (e.g. confidence intervals)
☐	☒	For null hypothesis testing, the test statistic (e.g. *F*, *t*, *r*) with confidence intervals, effect sizes, degrees of freedom and *P* value noted<br>*Give P values as exact values whenever suitable.*
☒	☐	For Bayesian analysis, information on the choice of priors and Markov chain Monte Carlo settings
☒	☐	For hierarchical and complex designs, identification of the appropriate level for tests and full reporting of outcomes
☒	☐	Estimates of effect sizes (e.g. Cohen's *d*, Pearson's *r*), indicating how they were calculated

Our web collection on statistics for biologists contains articles on many of the points above.

Software and code

Policy information about availability of computer code

Data collection	Sequencing was performed on Illumina NextSeq sequencing platform with NextSeq Control Software v.4.<br>ELISpot data were collected with AID ELISpot 7.0 and flow cytometry data were collected by BD FACSDiva V9.0 or Attune™ NxTsoftware v3.2.1
Data analysis	BCL files were converted to FASTQ format using bcl2fastq v2.20.0.422 (Illumina). FASTQ files were aligned to human genome hg19 using STAR v2.6.1d. Reads were counted using featureCounts (subread v2.0.0). The resulting counts matrix was analyzed in R v4.3.1 using Seurat v4.0.1. Data integration was carried out using Harmony v.1.0. TCR sequences were reconstructed from SmartSeq2 scRNAseq FASTQ files using MiXCR v.3.0.13 and imported into R using immunarch v0.9. Circos plots were generated using circlize package (v0.4.12) and all other plots using ggplot2 (v3.5.1) and ggpubr (v.0.4.0).<br>PeptGen software (http://www.hiv.lanl.gov/content/sequence/PEPTGEN/peptgen.html) was used to design 257 15- to 18-mer peptides overlapping by ten amino acid residues and spanning the full proteome of MPXV A9, F3, E12, D10 and D1 .<br>Flow cytometry data were analyzed with FlowJoTM v.10.9.0 software for Mac. IBM SPSS Statistics 25 was used for statistical analysis

For manuscripts utilizing custom algorithms or software that are central to the research but not yet described in published literature, software must be made available to editors and reviewers. We strongly encourage code deposition in a community repository (e.g. GitHub). See the Nature Portfolio guidelines for submitting code & software for further information.

Data

Policy information about availability of data

All manuscripts must include a data availability statement. This statement should provide the following information, where applicable:

- Accession codes, unique identifiers, or web links for publicly available datasets
- A description of any restrictions on data availability
- For clinical datasets or third party data, please ensure that the statement adheres to our policy

The single-cell RNAseq raw data can be found at ArrayExpress accession E-MTAB-14244

Research involving human participants, their data, or biological material

Policy information about studies with human participants or human data. See also policy information about sex, gender (identity/presentation), and sexual orientation and race, ethnicity and racism.

Reporting on sex and gender	Sex is provided for participants in cohort characteristics tables. Samples were all males and as such, no further adjustment on sex was carried out
Reporting on race, ethnicity, or other socially relevant groupings	NA
Population characteristics	13 individuals were recruited following recovery from Mpox infection. All participants were male, aged 23-60 years old. 3/13 had co-morbidities, including two with well-controlled HIV infection. In addition, 10 control individuals who are naïve to MPXV infection were studied in parallel. Moreover, a further 20 individuals were recruited following vaccination with the MVA-BN vaccine, all the individuals are male and aged between 26-77 years old . Participant characteristics are summarized in Table 1 and Supplementary Table 3.
Recruitment	The vaccination cohort study was a prospective observational study of the immune responses to the MVA-BN vaccination. Individuals attending the vaccination clinic in Oxford, UK and receiving the MVA-BN vaccine were invited to participate in this study. Individuals were invited to participate in the ISARIC Clinical Characterisation Protocol (REC: 13/SC/0149) study if they had presented with a clinical syndrome compatible with the 2022 mpox outbreak, and had one or more molecular tests performed on throat, rectal, skin or vesicle swab(s) positive for Clade II MPXV infection.
Ethics oversight	Individuals were invited to participate in the ISARIC Clinical Characterisation Protocol (REC: 13/SC/0149). Vaccination study was approved by the UK NHS Ethics committee (London – Surrey Borders Research REC: 22/PR/1425

Note that full information on the approval of the study protocol must also be provided in the manuscript.

Field-specific reporting

Please select the one below that is the best fit for your research. If you are not sure, read the appropriate sections before making your selection.

☒ Life sciences ☐ Behavioural & social sciences ☐ Ecological, evolutionary & environmental sciences

For a reference copy of the document with all sections, see nature.com/documents/nr-reporting-summary-flat.pdf

Life sciences study design

All studies must disclose on these points even when the disclosure is negative.

Sample size	13 individuals recovered from Mpox, 10 unexposed healthy control individuals and 20 unexposed but MVA-BN vaccinated control individuals. This was a discovery project for characterizing MPXV-specific T cell response in an unpublished UK population, and so predictive sample size calculations were challenging. Samples sizes were based on maximal available samples sets where detailed clinical and serological data were also available, and aligned well with our previously published data in other settings, including influenza, HIV and SARS-CoV-2(Peng et al NI 2019, Lee et al, JCI 2008; Zhang et al, NC 2013; Zhao et al, AJCCM 2012)
Data exclusions	For ELISpot assay, if negative control wells had >30 SFU per 10^6 PBMCs or positive control wells (PHA stimulation) were negative, the results were excluded from further analysis. There were no data excluded from ELISpot analysis. For AIMs assay, Mpox011 was excluded from analysis due to the high back ground at the negative control.
Replication	Samples analyzed in this study were from participants of a cohort study and samples were analyzed on individual study participants. Ex vivo ELISpot included duplicated wells, other experiments did not include replicates as all participants and data points are unique. For in vitro assays, results shown are always two or more independent experiments in which every repeat gave similar results.
Randomization	Randomization was not appropriate for this study of immune response in Mpox convalescent individuals, with no associated therapeutic intervention
Blinding	Blinding was not appropriate for this study of immune response in Mpox convalescent individuals, with no associated therapeutic intervention

Reporting for specific materials, systems and methods

We require information from authors about some types of materials, experimental systems and methods used in many studies. Here, indicate whether each material, system or method listed is relevant to your study. If you are not sure if a list item applies to your research, read the appropriate section before selecting a response.

Materials & experimental systems

n/a	Involved in the study
☐	☒ Antibodies
☐	☒ Eukaryotic cell lines
☒	☐ Palaeontology and archaeology
☒	☐ Animals and other organisms
☒	☐ Clinical data
☒	☐ Dual use research of concern
☒	☐ Plants

Methods

n/a	Involved in the study
☒	☐ ChIP-seq
☐	☒ Flow cytometry
☒	☐ MRI-based neuroimaging

Antibodies

Antibodies used

Marker	Fluorophore	Supplier	Cat number	Clonotype	Lot number	Dilution
anti-CD28/anti-CD49d		BD Biosciences	347690	L293		1µg/mL
Anti CD40 Ab		Miltenyi	130-094-133	HB14	520205616	0.5µg/mL
CD14	BV510	BioLegend	301842	M5E2	B349984	1:33
CD16	BV510	BioLegend	302048	3G8	B386487	1:33
CD19	BV510	BioLegend	302242	HIB19	B370761	1:33
CD3	BUV395	BD Biosciences	564001	SK7	1194980	1:50
CD8	PerCP-Cy5.5	BD Biosciences	565310	SK1	2059783	1:33
CD8	FITC	BD Biosciences	345772	SK1	2355287	1:33
IFNg	PE Cy7	BD Biosciences	557643	B27	1229911	1:50
TNFa	APC	Thermofisher Scientific	17-7349-82	MAb11	2330486	1:200
CD107a	PE	BD Biosciences	555801	H4A3	1243758	1:20
CCR7	BV421	Biolegend	353208	G043H7	B361376	1:33
CD27	PE-Cy7	Biolegend	356412	M-T271	B368443	1:50
CD27	BUV496	BD Biosciences	750168	L128	4004752	1:50
CX3CR1	BV711	Biolegend	341630	2A9-1	B376575	1:33
CX3CR1	BV650	Biolegend	341626	2A9-1	B391137	1:33
KLRG1	AF488	Thermofisher Scientific	53-9488-42	13F12F2	2354456	1:33
KLRG1	APC	Thermofisher Scientific	17-9488-42	13F12F2	2489376	1:33
CD45RA	APC-H7	BD Biosciences	560674	HI100	3115564	1:33
CD57	BV785	BioLegend	393330	QA17A04	B376899	1:33
CD4	BV711	BD Horizon	563028	SK3	3108277	1:350
OX40	BV421	Biolegend	350013	Ber-ACT35	B373164	1:33
CD137	APC-Cy7	Biolegend	309829	4B4-1	B382629	1:33
CD69	PE	BD pharmingen	555531	FN50	2301728	1:20
CD40L	PE-Cy7	Thermofisher Scientific	25-1548-42	24-31	2604290	1:20
CD3	Percp5.5	Biolegend	344808	Sk7	B372626	1:33
CD3	FITC	BD pharmingen	345764	Sk7	3174326	1:25
CD8	BV421	Biolegend	344748	SK1	B351415	1:50
IL-2	BV421	Biolegend	500328	MQ1-17H12	B320878	1:33
CD19	BV421	Biolegend	302234	HIB19	B275425	1:33
MIP1b	APC-H7	BD Biosciences	561280	D21-1351	2146486	1:33
CD3	BUV805	BD Biosciences	612895	UCHT1	325595	1:66
CD44	BUV496	Thermofisher Scientific	364-0441-80	1M7	2971239	1:100
CD8	BUV395	BD Biosciences	563795	RPA-T8	268343	1:50
CD49d	BV786	BD Biosciences	744588	9F10	4145307	1:100
CD45RA	BV711	Biolegend	304138	HI100	B428070	1:100
Granzyme A	PerCP-Cy5.5	Biolegend	507216	CB9	B362431	1:33
CD29	PE-Dazzle	Biolegend	303032	TS2/16	B376681	1:50
Granulysin	AF647	Biolegend	348006	DH2	B404190	1:33
Perforin	AF488	Biolegend	353320	B-D48	B365214	1:50
CD69	APC-Cy7	Biolegend	310914	FN50	B284262	1:33

Validation

All antibodies used in this study are commercially available. Antibodies used in a specific species or application have been appropriately validated by manufacturers for that application and this information is provided on their website and product

information datasheets listed below. All antibodies described here have been further optimized for an appropriate concentration by testing several dilutions.
CD14-BV510 https://www.biolegend.com/en-us/products/brilliant-violet-510-anti-human-cd14-antibody-14983
CD16-BV510 https://www.biolegend.com/en-us/products/brilliant-violet-510-anti-human-cd16-antibody-8003
CD19-BV510 https://www.biolegend.com/en-us/products/brilliant-violet-510-anti-human-cd19-antibody-8004
CD19-BV421 https://www.biolegend.com/en-gb/products/brilliant-violet-421-anti-human-cd19-antibody-7144
TNFa-APC https://www.thermofisher.com/antibody/product/TNF-alpha-Antibody-clone-MAb11-Monoclonal/17-7349-82
CD107a-PE https://www.bdbiosciences.com/eu/applications/research/intracellular-flow/intracellular-antibodies-and-isotype-controls/anti-human-antibodies/pe-mouse-anti-human-cd107a-h4a3/p/555801
CD3-FITC https://www.bdbiosciences.com/sg/reagents/clinical/reagents/single-antibodies/cd3-fitc-sk7-also-known-as-leu-4/p/345763
CD3-PerCP Cy5.5 https://www.biolegend.com/en-gb/products/percp-cyanine5-5-anti-human-cd3-antibody-6932
CD8 PerCP cy5.5 https://www.bdbiosciences.com/sg/reagents/research/antibodies-buffers/immunology-reagents/anti-non-human-primate-antibodies/cell-surface-antigens/percp-cy55-mouse-anti-human-cd8-sk1/p/565310
CD8-FITC https://www.bdbiosciences.com/en-gb/products/reagents/flow-cytometry-reagents/clinical-diagnostics/single-color-antibodies-asr-ivd-ce-ivd/cd8-fitc.345772
CD8-BV421 https://www.biolegend.com/en-gb/products/brilliant-violet-421-anti-human-cd8-antibody-13512
MIP1β-APC-H7 https://www.bdbiosciences.com/sg/applications/research/intracellular-flow/intracellular-antibodies-and-isotype-controls/anti-human-antibodies/apc-h7-mouse-anti-human-mip-1-d21-1351/p/561280
IFNγ-PE-Cy7 https://www.bdbiosciences.com/en-gb/products/reagents/flow-cytometry-reagents/research-reagents/single-color-antibodies-ruo/pe-cy-7-mouse-anti-human-ifn.557643
IL-2-BV421 https://www.biolegend.com/en-gb/products/brilliant-violet-421-anti-human-il-2-antibody-7148
CCR7-BV421 https://www.biolegend.com/en-gb/products/brilliant-violet-421-anti-human-cd197-ccr7-antibody-7497
CD27-PE-Cy7 https://www.biolegend.com/en-gb/products/pe-cyanine7-anti-human-cd27-antibody-8640
CD27-BUV496 https://www.bdbiosciences.com/en-gb/products/reagents/flow-cytometry-reagents/research-reagents/single-color-antibodies-ruo/buv496-mouse-anti-human-cd27.750168
CX3CR1-BV711 https://www.biolegend.com/en-gb/products/brilliant-violet-711-anti-human-cx3cr1-antibody-17965
CX3CR1-BV650 https://www.biolegend.com/en-gb/products/brilliant-violet-650-anti-human-cx3cr1-antibody-14960
KLRG1-AF488 https://www.thermofisher.com/antibody/product/KLRG1-Antibody-clone-13F12F2-Monoclonal/53-9488-42
KLRG1-APC https://www.thermofisher.com/antibody/product/KLRG1-Antibody-clone-13F12F2-Monoclonal/17-9488-42
CD45RA-APC-H7 https://www.bdbiosciences.com/en-gb/products/reagents/flow-cytometry-reagents/research-reagents/single-color-antibodies-ruo/apc-h7-mouse-anti-human-cd45ra.560674
CD57-BV785 https://www.biolegend.com/en-gb/products/brilliant-violet-785-anti-human-cd57-recombinant-antibody-19341
CD4-BV711 https://www.bdbiosciences.com/en-gb/products/reagents/flow-cytometry-reagents/research-reagents/single-color-antibodies-ruo/bv711-mouse-anti-human-cd4.563028
OX40-BV421 https://www.biolegend.com/en-gb/products/brilliant-violet-421-anti-human-cd134-ox40-antibody-7335
CD137-APC-Cy7 https://www.biolegend.com/en-gb/products/apc-cyanine7-anti-human-cd137-4-1bb-antibody-13508
CD69-PE https://www.bdbiosciences.com/en-gb/products/reagents/flow-cytometry-reagents/research-reagents/single-color-antibodies-ruo/pe-mouse-anti-human-cd69.555531
CD40L-PE-Cy7 https://www.thermofisher.com/antibody/product/CD154-CD40-Ligand-Antibody-clone-24-31-Monoclonal/25-1548-42
CD3-BUV805 https://www.bdbiosciences.com/en-gb/products/reagents/flow-cytometry-reagents/research-reagents/single-color-antibodies-ruo/buv805-mouse-anti-human-cd3.612895
CD44-BUV496 https://www.thermofisher.com/antibody/product/CD44-Antibody-clone-IM7-Monoclonal/364-0441-80
CD8-BUV395: https://www.bdbiosciences.com/en-gb/products/reagents/flow-cytometry-reagents/research-reagents/single-color-antibodies-ruo/buv395-mouse-anti-human-cd8.563795
CD49d-BV786 https://www.bdbiosciences.com/en-gb/products/reagents/flow-cytometry-reagents/research-reagents/single-color-antibodies-ruo/bv786-mouse-anti-human-cd49d.744588
CD45RA-BV711 https://www.biolegend.com/en-gb/products/brilliant-violet-711-anti-human-cd45ra-antibody-7937
Granzyme A-PerCP-Cy5.5 https://www.biolegend.com/en-gb/products/percp-cyanine5-5-anti-human-granzyme-a-antibody-5609
CD29-PE-Dazzle https://www.biolegend.com/en-gb/products/pedazzle-594-anti-human-cd29-antibody-18573
Granulysin-AF647 https://www.biolegend.com/en-gb/products/alexa-fluor-647-anti-human-granulysin-antibody-6587
Perforin-AF488 https://www.biolegend.com/en-gb/products/alexa-fluor-488-anti-human-perforin-antibody-16596
CD69-APC-Cy7 https://www.biolegend.com/en-gb/products/apc-cyanine7-anti-human-cd69-antibody-1917

Eukaryotic cell lines

Policy information about cell lines and Sex and Gender in Research

Cell line source(s)	All the EBV-transformed B cell lines were established in the lab.
Authentication	EBV transformed B cell lines expressing CD19 were verified by flow cytometry staining.
Mycoplasma contamination	All the cell lines were tested negative for mycoplasma
Commonly misidentified lines (See ICLAC register)	No misidentified cell lines were used according to the version 11 of register of misidentified cell lines

Plants

Seed stocks

NA

Novel plant genotypes

NA

Authentication

NA

Flow Cytometry

Plots

Confirm that:

- [x] The axis labels state the marker and fluorochrome used (e.g. CD4-FITC).
- [x] The axis scales are clearly visible. Include numbers along axes only for bottom left plot of group (a 'group' is an analysis of identical markers).
- [x] All plots are contour plots with outliers or pseudocolor plots.
- [x] A numerical value for number of cells or percentage (with statistics) is provided.

Methodology

Sample preparation

For cell sorting, cryopreserved PBMCs were thawed and rested overnight in R10 at 37oC. On the second day, PBMCs were stained with PE-conjugated HLA-A2 ILD-specific Tetramer. Live/Dead fixable Aqua dye (Invitrogen) was used to exclude non-viable cells from the analysis. Subsequently, cells were washed and stained with the following surface antibodies: CD3-FITC (BD Biosciences), CD8-PercP-Cy5.5, CD14-BV510, CD19-BV510 and CD16-BV510 (Biolegend). After the final wash, cells were resuspended in 500µl of PBS and 0.5% BSA (Sigma-Aldrich) solution and kept in dark at 4°C until flow cytometric acquisition. After exclusion of non-viable/CD19+/CD14+/CD16+ cells, CD3+ CD8+ Tetramer+ cells were sorted directly into 96-well PCR plates containing cell lysis buffer using a BD Fusion 1 or BD FACS Aria III (BD Biosciences). For AIMs assay, before co-culturing with 0.3x10^6 VACV-infected PBMCs, 1.2 x10^6 fresh or cryopreserved autologous PBMCs were blocked at 37°C for 15 min with 0.5 µg/ml of anti-CD40 monoclonal antibody, followed by the addition of anti-CD28/CD49a (BD) at a final concentration of 1µg/ml. Subsequently, cells were incubated at 37°C for 20-24 hours in 96-well U-bottom plates. The next day, PBMCs were resuspended in phosphate-buffered saline (PBS), incubated with BD human FC block and a LIVE/DEAD marker in the dark for 15 min and washed with PBS containing 5% FBS (FACS buffer).An antibody mix containing the rest of the surface antibodies in Brilliant Stain Buffer (BD) was added directly to cells and incubated for 50-60 min at 4°C in the dark. After surface staining, cells were washed twice with PBS containing 5% FBS. For intracellular cytokine staining (ICS), in vitro expanded T cell lines/ clones were co-cultured with autologous B cell lines loaded with peptides, or infected with Vacinnia virus/live virus at an appropriate E:T ratio, in the presence of GolgiPlug (brefeldin A, BD), GolgiStop (monensin, BD) and 5ul of PE-anti-CD107a (BD Biosciences) for 5hrs. Then a standard FACS staining was carried out. Briefly, dead cells were first labelled with LIVE/DEAD™ Fixable Aqua dye and then followed by surface antibody staining. Subsequently, Cytofix/CytopermTM kit (BD Biosciences) was used for permeabillizing the cells before staining the cells with antibodies against molecules expressed intracellularly. Finally, cells then be fixed with 1X cell fixing buffer. For CFSE-based killing assay, HLA-A*02:01-positive BCLs were infected with the VACV strain Lister at an MOI of 3 overnight, cells were washed and counted before being labelled with 0.5 µM carboxyfluoroscein succinimidyl (ThermoFisher). Subsequently, BCLs were then co-cultured with T cells at an E:T ratio of 2:1, 1:1 and 1:2 at 37°C for 6hrs. Samples were then stained with 7-AAD (eBioscience) and CD19-BV421 (BioLegend). For ex vivo phenotyping on CD8+Tetramer+ T cells, 2-6 X10^6 PBMCs were thawed and rested for 3hrs before staining with tetramer fat 37°C for 20mins. After washing with PBS, cells were first labelled with LIVE/DEAD™ Fixable Aqua dye and then followed by surface antibody staining. Subsequently, Cytofix/CytopermTM kit (BD Biosciences) was used for permeabillizing the cells before staining the cells with antibodies against molecules expressed intracellularly. Finally, cells then be fixed with 1X cell fixing buffer.

Instrument

Samples were sorted using BD Fusion 1 or BD FACS Aria III (BD Biosciences) or acquired at Thermo Fisher Attune™ NxT Flow Cytometer or BD LSRFortessa X50 (BD Biosciences)

Software

Data were collected using FACS DIVA (v9.0.1) or Attune™ NxTsoftware v3.2.1 and analyzed using FlowJoTM v.10 software for Mac.

Cell population abundance

All sorted samples were checked for after-sorting purity (>99%) and teramer+ T cells over 50 cells

Gating strategy

For all the experiments, cells were first gated on single Lymphocytes by a forward side scatter gate. On sorting for RNASeq, after excluding dead cells, CD14+, CD19+, and CD16+ cells, HLA-A2-ILD specific T cells were identified as CD3+CD8+Tetramer +. For intracellular cytokine staining (ICS), the cytokine positive/ negative population were gated according to corresponding negative controls, known as unstimulated samples (T cells co-cultured with target cells without virus infection): after excluding dead cells, cells then were gated into CD8+ T cells IFN-g+/-, TNFa+/-, MIP11ß+/- and CD107a+/- populations were

gated in consistence with the corresponding negative controls. For killing assay, live target cells were identified as CFSE+7-AAD- cells

☒ Tick this box to confirm that a figure exemplifying the gating strategy is provided in the Supplementary Information.
